# Artificial Intelligence–Driven Tools in Mental Health Service Delivery: A Scoping Review

**DOI:** 10.3390/healthcare14070943

**Published:** 2026-04-03

**Authors:** Yeshin Woo, Kibum Jung

**Affiliations:** 1 Department of Research & Development, Incheon Metropolitan Mental Health Welfare Center, Incheon 22106, Republic of Korea; 2 Department of Physical Therapy, Graduate School, Sahmyook University, Seoul 01795, Republic of Korea; 3 Department of Physical Therapy, Kangbuk Samsung Hospital, Seoul 03181, Republic of Korea

**Keywords:** artificial intelligence, mental health services, scoping review, implementation science, large language models, digital health

## Abstract

**Background**: Artificial intelligence (AI) holds transformative potential for mental health services. However, existing reviews have predominantly focused on algorithmic accuracy, with limited attention to how these technologies are implemented and integrated into real-world service delivery. This scoping review addresses this gap by examining the contexts in which AI technologies—including large language models (LLMs) and machine learning—are implemented, as well as the factors influencing their sustainable adoption within real-world mental health service systems. **Methods**: Following the established methodological framework, a systematic search (2015–2026) was conducted in PubMed and Scopus. Two independent reviewers screened an initial pool of 829 records using Zotero and Rayyan to minimize selection bias. Following title, abstract, and full-text screening based on predefined eligibility criteria, 26 studies focusing on real-world AI applications (e.g., clinical settings, community services, and case management) were included in the final synthesis. **Results**: The findings indicate a rapid acceleration in research, with 50% of included studies (*n* = 13) published since 2024. AI-driven decision support systems were the most prevalent (50%, *n* = 13), followed by predictive machine learning models (27%) and generative AI applications (15%). Most tools were designed for clinician use (77%) and implemented in hospital-based settings (46%). Although 46% of studies reported real-world implementation, more than half remained at the pilot stage. Notably, research emphasis has shifted from technical efficacy toward feasibility, and implementation contexts (*n* = 17). **Conclusion**: AI in mental health is transitioning from laboratory validation to real-world integration. However, the current landscape remains heavily centered on clinician workflows and screening functions, with limited expansion into community-based recovery and long-term prevention. To move beyond the pilot stage, future initiatives should prioritize seamless workflow integration and the application of structured ethical and implementation frameworks that support clinician–patient relationships. This review provides an evidentiary basis for advancing sustainable, AI-enhanced mental health service delivery.

## 1. Introduction

In the context of ongoing digital transformation, the role of Artificial Intelligence (AI) continues to expand rapidly. As the demand for mental health services surges, interest in leveraging AI technologies—such as Large Language Models (LLM), Machine Learning (ML), and Natural Language Processing (NLP)—are gaining attention as potential solutions to bridge the gap caused by workforce shortages, increasing workloads, and declining service quality. Advances in AI technologies are enhancing the quality of mental health services and providing novel approaches to digital service delivery. By collecting multifaceted data, algorithms support personalization, predict risks, and suggest specific interventions by identifying temporal trends in mental health changes [1,2,3,4].

A significant transformation in the field of mental health services is the transition from traditional reactive systems toward proactive prevention strategies. Previous studies have highlighted the role of ML algorithms in enabling such transitions through the use of real-time data. In particular, in the context of acute crises such as suicidal ideation, these technologies enable a shift from traditional approaches that identify crises only after they occur to predictive approaches that allow for early identification and preemptive intervention before a crisis develops [5,6]. Furthermore, AI is increasingly viewed as a key approach to addressing the growing mismatch between rising service demand and the limited expansion of the mental health workforce. Advanced technological interventions, including generative AI, have the potential to improve system efficiency and reduce long waiting times that previously extended over several months, thereby enabling the implementation of a just-in-time intervention paradigm [7,8].

The rapid advancement of generative artificial intelligence, led by large language models (LLMs), has further broadened the scope of AI applications in mental health services. Unlike traditional rule-based or predictive systems, LLM-based technologies enable more flexible and context-aware interactions through natural language conversations. These systems can substantially improve access to mental health support through conversational and user-centered interfaces, suggesting their potential to reduce barriers to care. Consequently, LLM-based tools are increasingly being explored as complementary resources that can support early intervention and self-management while augmenting existing mental health service systems.

Existing research has primarily focused on attitudes, readiness, and technology-centered evaluations, emphasizing model accuracy and algorithmic performance [9,10]. However, there is still a lack of systematic understanding of how AI is implemented and utilized within real-world service contexts, with limited empirical evidence of improved patient outcomes. To scale AI technologies from research to clinical practice, it is essential to move beyond technical accuracy and investigate them from an organizational and operational perspective. Consequently, this scoping review was conducted with an implementation-centered focus to examine how Artificial Intelligence is utilized within real-world mental health service delivery systems. Unlike prior reviews that primarily emphasize technological performance or user perceptions, this study systematically analyzes the implementation contexts, service functions, user groups, and stages of real-world deployment of AI-driven tools. By comprehensively reviewing the types of AI technologies, service functions, user types, and implementation stages applied over the past decade, this study aims to provide sustainable integration and practical adoption of AI-based mental health services.

This study aims to bridge this gap by conducting a comprehensive scoping review. Specifically, this review addresses the following research questions:

(1) What types of AI-driven tools have been implemented in mental health service delivery, and how have these evolved over time?
(2) What are the primary purposes and stages of implementation of AI-based mental health service systems?
(3) Which service functions are supported by AI systems in mental health services, and who are the primary intended users?
(4) What gaps exist in the current implementation and evaluation of AI-driven tools in mental health service settings?

## 2. Materials and Methods

### 2.1. Study Design

A scoping review is conducted to map key concepts and information related to a research question, as well as to examine the extent, range, and nature of evidence within a particular field. This approach is particularly effective when the research question is complex or when a comprehensive analysis has not been previously undertaken [11]. Moving beyond the technical effectiveness of AI, this research analyzes how AI is utilized in mental health services. Specifically, we focus on the social, organizational, and participatory conditions required to enable the practical application of AI in this field.

This scoping review was conducted in accordance with the PRISMA-ScR (Preferred Reporting Items for Systematic reviews and Meta-Analyses extension for Scoping Reviews) guidelines. We adopted the methodological framework proposed by Arksey and O’Malley, which consists of five stages; (1) identifying the research questions, (2) identifying relevant studies, (3) study selection, (4) charting the data, and (5) collating, summarizing, and reporting the results.

A review protocol was entered into the Open Science Framework (OSF) database (Registry number: y7rdp, Link: https://osf.io/8gue4/overview (accessed on 5 March 2026)).

### 2.2. Search Strategy

A systematic literature search was conducted in PubMed and Scopus to ensure broad coverage of biomedical, mental health, and interdisciplinary research, including emerging AI-related studies. The search was conducted in January 2026 and was limited to articles published in English between 2016 and 2026. Relevant studies at the intersection of mental health services, artificial intelligence, and service delivery were identified.

The search strategy combined Medical Subject Headings (MeSH) in PubMed with free-text keywords in both databases. Field tags such as [Title/Abstract] in PubMed and TITLE-ABS-KEY in Scopus were applied to ensure comprehensive retrieval of relevant records. Detailed search strategies, including full search strings and applied filters for each database, are provided in Table 1. The search strategy was intentionally designed to be broad to maximize sensitivity, particularly given the variability in terminology used to describe implementation in mental health AI research. As a result, some studies that did not meet the specific inclusion criteria were retrieved and subsequently excluded during the screening process.

### 2.3. Eligibility Criteria

This study specifically targets research where AI technology is implemented as a practical tool in real-world mental health services. All publications were systematically screened and selected based on predefined inclusion and exclusion criteria. Textbox 1 presents inclusion and exclusion criteria, which were developed to optimize the ability to address the research question. Selected articles were screened first at the title and abstract level. The resulting set of articles were screened in full using the same process. Article screening was conducted usingZotero (version 7.0.30) and Rayyan (version 1.7).

Inclusion criteria

- published in English with the full text available
- real-world implementation
- AI technology must be utilized by real users

(e.g., clinicians, patients, or practitioners)

- psychiatric or community-based mental health settings

(e.g., outpatient/inpatient clinics, helplines, crisis lines, or case management)

Exclusion criteria

- No real-world implementation
- Non-AI or non-mental health focus
- Non-empirical study types (e.g., survey, review)

### 2.4. Study Selection and Data Extraction

The study selection process was conducted from 5 January to 12 January 2026. Bibliographic data from the identified studies were exported from each database and managed using Zotero (version 7.0.30) and Rayyan (version 1.7). To ensure objectivity and minimize selection bias, two independent reviewers participated in the entire selection process. They collaboratively established the data collection criteria and items, and independently performed the search to ensure no relevant studies were omitted by cross-checking the results. Any discrepancies regarding whether a study met the inclusion or exclusion criteria were resolved through mutual discussion and consensus.

**Figure 1:**
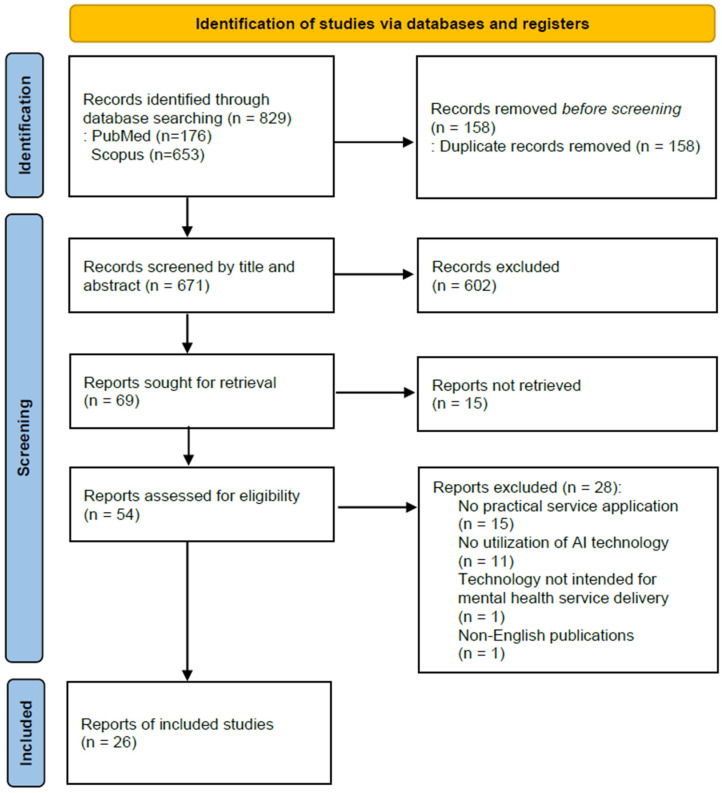
PRISMA Flow Chart. PRISMA (Preferred Reporting Items for Systematic Reviews and Meta-Analyses) 2020 flow diagram illustrating the study selection process. The systematic search was conducted across PubMed and Scopus databases. A total of 26 studies met the inclusion criteria for the final qualitative synthesis.

A total of 829 studies were initially identified (176 from PubMed and 653 from Scopus). In the first phase of screening, 158 duplicate records were removed. During the second phase, the titles and abstracts were screened for relevance to the research topic; at this stage, 602 studies that did not focus on the intersection of AI, mental health, and implementation were excluded. In the third phase, a full-text review was performed, leading to the exclusion of further studies for the following reasons: unavailable full-text (*n* = 15), no practical service application (*n* = 15), no utilization of AI technology (*n* = 11), technology not intended for mental health service delivery (*n* = 1), and non-English publications (*n* = 1). Through this rigorous process, 26 studies were ultimately selected for the final analysis after a final cross-verification by the two reviewers (Figure 1, Table A1 and Table A2).

### 2.5. Data Items and Charting

Data were extracted and synthesized following Stages 4 and 5 of Arksey and O’Malley’s (2005) [11] framework. In Stage 4, we used a standardized data charting form (Table 2) to extract key information from the 26 selected studies. The data items were categorized into three dimensions: (1) General Study Characteristics (Year, Study Design, Age Group), (2) AI Technological Configuration (AI Type, Data Sensitivity), and (3) Service Delivery Context (Service Function, Primary User, Setting, Implementation Stage, Study Focus). Data extraction and categorization were guided by a predefined extraction and mapping framework (Table 2). Each study was systematically coded across multiple dimensions. To enhance methodological transparency, explicit criteria were applied for each category. For example, service settings were classified as clinical, community-based, digital-only, or hybrid based on the primary context of implementation; user types were defined according to the main intended users of the AI system; and implementation stages were categorized based on the level of real-world deployment (e.g., pilot, implemented, or scale-up).

To ensure reliability, two researchers independently reviewed the extracted data, and any discrepancies were resolved through discussion and consensus. In Stage 5, the results were synthesized using a combination of numerical summaries and thematic mapping, with key findings presented in tables and figures aligned with the research questions.

### 2.6. Ethical Considerations

No ethics approval was applied for since the study involved only a review of published data. There are no human participations in this article and informed consent is not required.

## 3. Results

### 3.1. Characteristics of Included Studies and Implementation Contexts

Table 3 provides an overview of the key characteristics of the included studies, including AI type, service setting, user type, implementation stage, and outcome focus. The studies demonstrate diverse applications of AI technologies across clinical and community settings, targeting both clinicians and patients. The included studies demonstrated diverse real-world applications of AI technologies across clinical and community settings, targeting both clinicians and patients. Many interventions focused on improving service efficiency, such as reducing wait times and administrative burden, while others showed potential for enhancing clinical outcomes, including symptom reduction and treatment adherence. Additionally, several studies highlighted important implementation challenges, such as ethical concerns, data privacy, and integration into existing workflows. Table 3 provides a summary of key study characteristics to enhance readability, while detailed descriptions of each included study are presented in the Table A1 and Table A2.

### 3.2. Study Characteristics and Publication Trends

**Figure 2:**
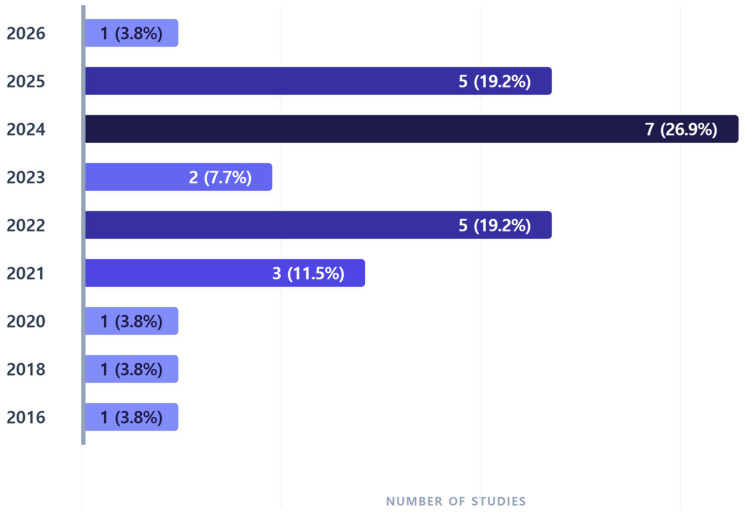
Annual Trends of Included Studies (N = 26). The horizontal bar chart illustrates the annual progression of research publications from 2016 to 2026. The color density provides a visual gradient with a narrower contrast range, representing the volume of studies where darker shades signify peak research activity.

A total of 26 studies published over the past decade were identified, and the annual publication trends are presented in Figure 2. From 2016 to 2020, one study (3.8%) was published each year. The number of publications increased to 3 studies (11.5%) in 2021. From 2022 onward, 5 studies (19.2%) were published, reaching a peak in 2024 with 7 studies (26.9%).

**Figure 3:**
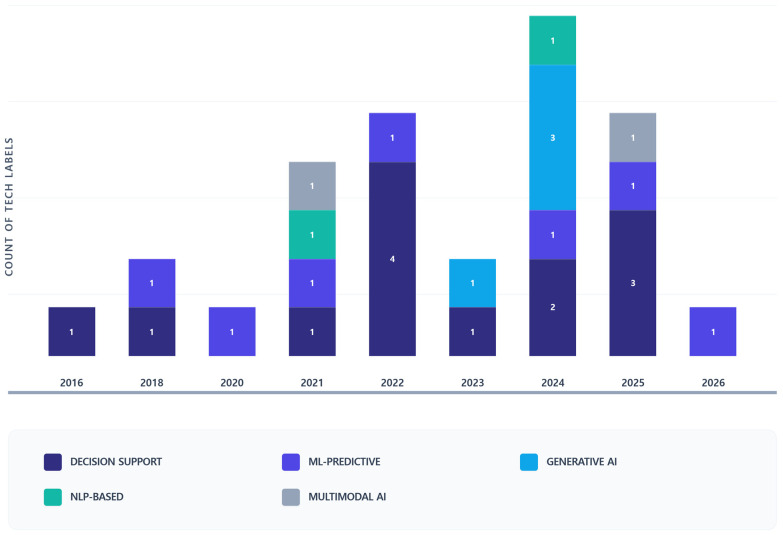
Annual Trends of AI Technology Types. The chart illustrates the technological evolution within the reviewed literature (N = 26). While traditional Decision Support and ML-Predictive models maintain a steady presence, there is a marked shift towards Generative AI starting in 2023. The diversity of AI applications reached its peak in 2024, showing a significant rise in NLP and multimodal compared to earlier years. Counts represent the frequency of AI technology types across studies; a single study may contribute multiple counts if more than one technology is used.

The results of the analysis regarding the trends in AI technology types by year are as follows Figure 3. During the Initial Phase (2018–2020), technologies were primarily focused on Decision Support or Machine Learning-Predictive, with 4 studies (15.4%) identified in this period. In the Growth Phase (2021–2023), the utilization of Natural Language Processing (NLP) and Multi-Modal AI technologies began to emerge, encompassing 9 identified studies (34.6%). Subsequently, in the Expansion Phase (2024–2026), Generative AI has rapidly risen to prominence, with a significant concentration of research on both Generative AI and Decision Support. In summary, the diversity of technologies has increased sharply since 2021. Due to the active use of Generative AI and NLP in 2024, more than 50% of the total research has been concentrated within the last three years.

### 3.3. AI Technologies and Functional Distribution

**Figure 4:**
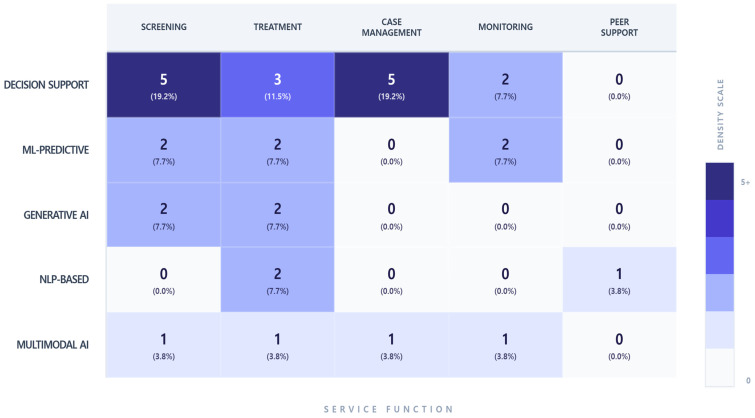
Distribution of AI Technology Types and Service Functions. This heat-map illustrates the intersection of AI technology types and their functional application within the 26 reviewed studies. Decision support systems are most frequently applied to screening and case management, while generative AI and NLP-based technologies are more commonly used in clinical treatment. Counts represent the number of occurrences of AI technologies across service functions; a single technology may be counted multiple times if applied to multiple functions within a study.

When analyzed by AI technology type used in mental health services, Decision Support systems (*n* = 15) and ML-based predictive models (*n* = 6) were the most frequently reported in Figure 4. From a functional perspective, Decision Support systems were most commonly applied to Screening and Assessment (19.2%) and Case Management (19.2%), followed by Treatment and Intervention (11.5%) and Monitoring and Follow-up (7.7%). ML-based predictive models were applied comparably across Screening and Assessment, Treatment and Intervention, and Monitoring and Follow-up (7.7% each).

### 3.4. Distribution of Implementation Stages Across Service Settings

**Figure 5:**
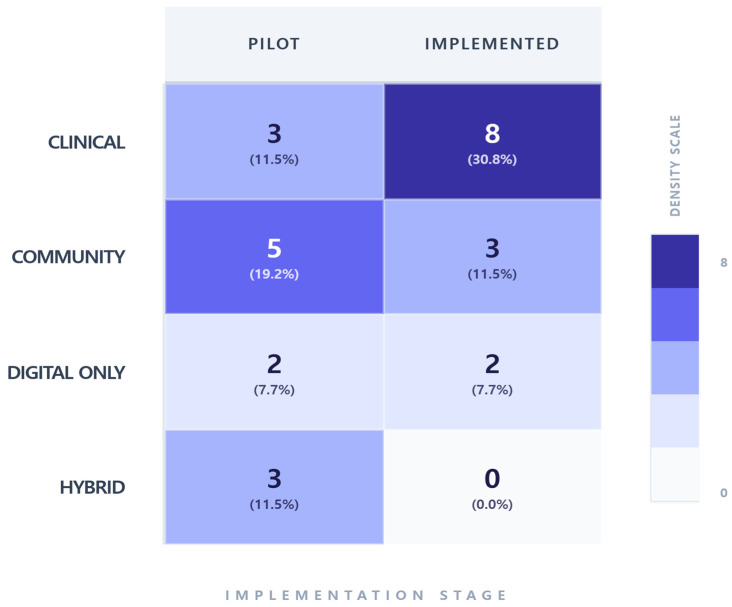
Implementation Stage by Settings. The heat-map illustrates the distribution of 26 studies across various service settings and implementation stages. The clinical setting shows the highest implementation maturity (*n* = 8, 30.8%), whereas hybrid and community settings exhibit a higher concentration of pilot-phase research. Values represent the absolute study count and the percentage relative to the total sample.

The results of the analysis of implementation stages according to AI service deployment settings are presented in Figure 5. When examined by implementation stage, 13 of the 26 studies (50.0%) reported AI applications that were implemented in real-world practice, while the remaining 13 studies (50.0%) were conducted at the pilot stage. Regarding deployment settings, clinical environments accounted for 11 studies (42.3%), followed by community settings in 8 studies (30.7%), digital-only settings in 4 studies (15.4%), and hybrid settings in 3 studies (11.5%). When implementation stages were examined by deployment setting, clinical environments showed a higher number of real-world implementation cases, with 8 studies (30.8%). In contrast, community settings more frequently reported pilot-stage, with 5 studies (19.2%). In digital-only settings, pilot-stage and real-world implementation cases were equally represented, with 2 studies each (7.7%). In hybrid settings, all identified AI services were at the pilot stage, comprising 3 studies (11.5%), and no cases of real-world implementation were identified.

### 3.5. Service Functions and Primary Users

Service functions supported by AI technologies and their primary intended users are summarized in Table 4. Overall, AI technologies were predominantly applied to support clinicians across multiple service functions. Case management and Screening/Assessment functions were most frequently clinician-facing, each reported in 9 studies (34.6%). Treatment/Intervention support for clinicians was also common, appearing in 8 studies (30.8%), followed by Monitoring/Follow-up function in 6 studies (23.1%). Patient-facing applications supported by AI technologies were less frequently reported and were primarily concentrated in treatment or intervention functions (*n* = 5, 19.2%). In contrast, no patient-facing applications were identified for case management or Screening/Assessment. Monitoring/Follow-up functions targeting patients were reported in only 2 studies (7.7%). Applications supported by AI technologies and designed for peer workers and service managers or administrators were relatively uncommon. Peer worker-focused applications were most often associated with Case management (*n* = 3, 11.5%) and Treatment/Intervention (*n* = 2, 7.7%). whereas Manager/Administrator appeared infrequently and were limited to Case management, Screening/Assessment, and Monitoring/Follow-up functions (each *n* = 1–2, 3.8–7.7%).

### 3.6. Distribution of Implementation Stages Across Service Functions

**Figure 6:**
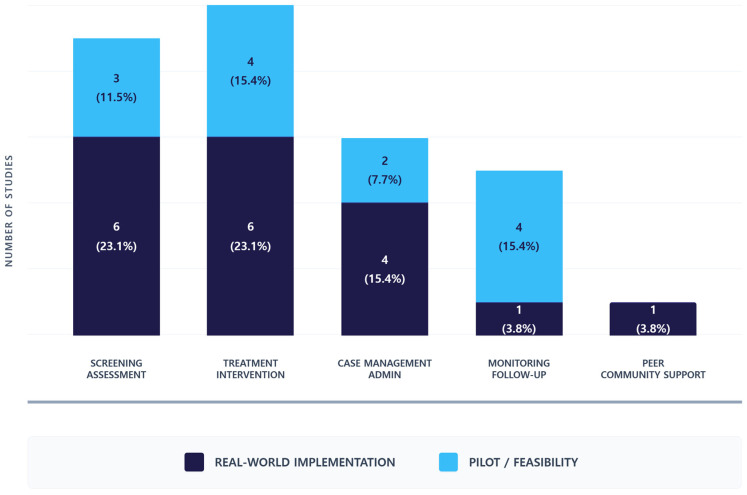
AI Implementation Maturity by Service Function. Values within the bars represent absolute counts and the percentage relative to the total number of studies. Because several studies addressed multiple functions simultaneously, the cumulative count across functions exceeds the total sample size. The analysis indicates that while treatment and screening are increasingly moving towards real-world application, functions like monitoring and peer support largely remain at the pilot phase.

The results of the analysis of service functions supported by AI technologies are presented in Figure 6. At the implementation stage, AI technologies were primarily applied to functions related to initial service assessment and treatment/intervention. Screening/Assessment and Treatment/Intervention were the most frequently reported functions, with 6 studies each (23.1%), followed by Case Management in 4 studies (15.4%). In contrast, at the pilot stage, AI technologies were most commonly used in the context of treatment/intervention and ongoing service monitoring, with Monitoring and Follow-up and Treatment/Intervention each reported in 4 studies (15.4%).

### 3.7. Diversity, Equity, and Bias Consideration

As summarized in Table 5, consideration of diversity, equity, and algorithmic bias was limited. Of the 26 studies, the majority (*n* = 11) did not report any discussion related to bias or equity (Level 0), beyond basic demographic descriptions. A larger proportion of studies (*n* = 11) included conceptual mentions of potential bias or generalizability concerns (Level 1), typically within the limitations section, but did not conduct empirical analyses. Only a small subset of studies (*n* = 4) performed explicit subgroup or bias evaluations (Level 2), such as examining differences in outcomes or model performance across demographic groups. These findings indicate that while awareness of bias is emerging, systematic evaluation remains limited in real-world AI applications in mental health services.

## 4. Discussion

In the early stage (2016–2020), artificial intelligence was primarily used for decision support or machine learning–based predictive technologies. During this period, AI was largely perceived in clinical settings not as an independent therapeutic tool but as a supportive tool that assisted clinicians’ decision-making processes. In the growth stage (2021–2023), the use of natural language processing (NLP) and multimodal technologies gradually emerged, and in the expansion stage (2024–2026), generative AI technologies rose rapidly. In particular, studies related to generative AI and decision support were reported more frequently during this period. This study also identified cases demonstrating a transition from predictive machine learning models that simply estimate risk based on data to conversational AI systems capable of understanding users’ emotional contexts through real-time interactions and providing adaptive feedback. Stroud et al. (2025) [14] implemented adjunctive cognitive behavioral therapy through a chatbot interface. Farrand et al. (2024) [20] developed a conversational agent using natural language processing to automate low-intensity cognitive behavioral therapy (CBT) techniques for worry management. Rollwage et al. (2022, 2023) [8,33] employed conversational AI chatbots to support patient self-referral and initial assessment processes, demonstrating improvements in recovery rates and cost-effectiveness. Sels et al. (2021) [5] utilized an open-source platform with a conversational interface to conduct ecological momentary assessments (EMA), asking patients questions in real time and monitoring and predicting suicidal ideation. These technological advancements have also led to structural changes in clinical workflows. In particular, routine tasks such as screening, monitoring, and initial assessment are increasingly being supported or partially automated by AI systems, resulting in task shifting from clinicians to AI-assisted processes. Consequently, clinicians are taking on more supervisory and interpretive roles within AI-enabled service environments.

Taken together, AI technologies appear to be evolving beyond simple risk prediction tools toward integrated service platforms in which clinicians, service users, and AI systems interact. Across technological categories, machine learning models have primarily been used for risk prediction [7,23], decision-support systems for augmenting clinical decision-making [13,14,21], and large language model–based tools for enabling interactive, user-facing interventions [8,19,20,33]. Such technological developments are also influencing the way mental health services are delivered. Accordingly, the roles of both professionals and users within AI-enabled service delivery systems are changing. Whereas AI tools were previously used primarily by professionals, there is an increasing trend toward self-guided interventions in which users directly interact with AI systems to manage their own mental health. Danieli et al. (2021) [28] further highlighted the role of users as active agents in service delivery, emphasizing participatory design approaches in which users are involved not only as recipients but also as contributors to the service design process.

The findings of this review indicate that AI technologies have been widely utilized to improve the efficiency of early screening and diagnostic processes. These findings also suggest that AI has the potential to enhance service accessibility by reducing barriers to care, particularly through digital and self-guided interventions. These findings suggest that automated evaluation processes based on large-scale data can play an important role in the early stages of mental health services [8]. Across technological categories, decision support systems have been consistently used throughout the study period, while the proportion of generative AI and NLP-based technologies has increased notably since 2024. This trend indicates the growing feasibility of real-time interventions delivered through chatbots or mobile applications. Moreover, AI technologies have been proposed as a potential solution to address shortages in human resources within public mental health services. However, high detection rates do not necessarily translate into sustained treatment engagement or long-term recovery outcomes [25]. Several structural and systemic barriers may explain this gap between detection and long-term outcomes. These include limited interoperability between healthcare systems, which constrains data sharing and continuity of care; the absence of sustainable reimbursement or business models that support long-term use of AI technologies; and variability in patients’ digital health literacy, which may affect engagement with AI-based interventions. In particular, integration with rehabilitation infrastructures that support patient independence [27] and preventive self-management functions [31] remain in early stages of development. Therefore, future technological development should extend beyond risk identification to support comprehensive service models that address recovery and prevention across the entire life course of patients.

With regard to the implementation stage of AI services, the included studies were relatively evenly distributed between pilot studies and real-world implementation studies. This suggests that AI technologies are gradually moving beyond experimental validation toward evaluation within real service environments. A notable strength of this review is that it includes a substantial number of studies conducted in real-world settings. In particular, more than 73% of studies conducted in clinical environments were found to be in the implementation stage, indicating that AI technologies are being adopted relatively stably within structured medical infrastructures. In contrast, approximately 62.5% of studies conducted in community settings were in the pilot stage, suggesting that these environments are still preparing for broader implementation. Studies conducted in hybrid settings were all at the pilot stage, indicating that integrated online–offline intervention models remain largely experimental. Overall, these findings suggest that AI research in mental health services is currently at a transitional stage, moving from pilot studies that examine technological feasibility toward implementation studies that evaluate real-world service outcomes. From a managerial perspective, these findings imply that AI implementation may significantly reshape organizational workflows, cost structures, and workforce roles. For example, prior studies have demonstrated cost efficiency, with estimates ranging from £103 to £207 per additional recovery, suggesting the potential for scalable and economically sustainable service models [33].

At the same time, the literature also highlights concerns regarding the adoption of AI technologies in mental health services. In addition, concerns regarding clinical safety, including the risk of inappropriate or unsafe recommendations generated by AI systems, have been increasingly emphasized. In particular, practical challenges such as clinician adoption, the need for training and digital literacy, interoperability with existing electronic health record systems, and alignment with clinical workflows remain critical barriers to large-scale implementation [14,31,32]. Although AI has the potential to improve service efficiency, ethical concerns such as algorithmic bias that may produce unequal outcomes for minority groups or potential threats to patient autonomy have been identified as significant barriers to adoption [12]. Some clinicians also express concern that AI systems may be misused as tools for monitoring professional performance rather than supporting clinical practice, while issues related to the protection of sensitive patient data remain a key concern [32]. Healthcare professionals have also raised concerns that excessive reliance on AI could lead to the erosion of clinical expertise, as well as uncertainty regarding legal responsibility in cases of incorrect algorithmic recommendations [14]. In practice, the use of AI technologies may also increase technological fatigue among patients and administrative complexity for clinicians [31], while limited health infrastructure in some settings poses additional challenges for system implementation [25]. Therefore, for AI technologies to be sustainably integrated into mental health services, strategies must go beyond technological advancement and address clinical workflow integration while establishing ethical trust in the use of AI systems.

### 4.1. Limitations

This study has several limitations. First, as this study was conducted as a scoping review, a formal methodological quality assessment of the included studies was not performed. Therefore, the strength of the evidence should be interpreted with caution.

Second, the literature search was limited to two databases (PubMed and Scopus), and did not include certain discipline-specific databases, such as PsycINFO and information technology–focused databases. This may have resulted in the omission of relevant studies, particularly those at the intersection of mental health and digital innovation.

Third, the included studies showed substantial heterogeneity in terms of AI technologies, service contexts, target populations, and outcome measures, which limited direct comparisons across studies. Finally, given the rapid evolution of AI technologies, newly emerging tools and evidence may not have been fully reflected within the review period. Fourth, many of the included studies were conducted at the pilot stage with relatively small sample sizes, and the lack of standardized outcome measures limits the generalizability and comparability of findings.

Furthermore, the limited consideration of diversity, equity, and algorithmic bias across the included studies represents an important limitation. Although some studies acknowledged the potential for bias, most did not conduct empirical subgroup analyses or evaluate differences in outcomes across demographic groups. Given that mental health outcomes and access to care vary across demographic and socioeconomic populations, this lack of evaluation restricts the ability to assess the fairness and generalizability of AI-driven interventions.

### 4.2. Future Directions

Future research should move beyond focusing solely on specific AI technologies or implementation stages and instead adopt structured implementation science approaches. In particular, applying standardized frameworks such as the Consolidated Framework for Implementation Research (CFIR) and RE-AIM will enable systematic evaluation of contextual factors, implementation processes, and long-term outcomes, including treatment continuity and recovery.

In addition, future studies should prioritize real-world evaluation beyond pilot settings, including large-scale implementation, long-term follow-up, and integration with existing healthcare systems. Greater attention is also needed to address ethical and safety considerations, including data privacy, algorithmic bias, and clinical accountability. In particular, future research should incorporate fairness evaluation frameworks, such as systematic subgroup analyses and bias mitigation strategies, to ensure equitable performance across diverse populations. As AI technologies become increasingly embedded in real-world mental health services, such efforts will be essential to prevent the reinforcement of existing health disparities.

Furthermore, research should explore how AI technologies can support community-based recovery, preventive care, and patient-centered service models, rather than focusing primarily on screening and clinician-centered workflows.

## 5. Conclusions

In the field of mental health, the application of AI has shifted beyond simple predictive models toward interactive services powered by large language models (LLMs). The focus of research has likewise expanded from accuracy validation to the assessment of real-world applicability, feasibility, and implementation outcomes. However, technological advancement does not necessarily translate into improvements in service quality. Persistent challenges remain, including data privacy, algorithmic bias, and the clinical validity. In addition, concerns related to clinical safety, accountability, and responsible use of AI systems must be addressed to ensure safe deployment in mental health services. Although predictive performance has improved, current AI models remain limited in fully replacing professional decision-making in clinical and community settings, particularly with respect to explainability and accountability.

Nevertheless, AI applications are demonstrating the potential to improve service efficiency and expand access to care, particularly through digital and scalable interventions. At the same time, the integration of AI into mental health services has important implications for organizational structures, workforce roles, and cost efficiency, highlighting the need for system-level adaptation and sustainable implementation strategies. This study contributes to the literature by moving beyond a technology- or performance-centered analysis and examining AI applications within the broader context of service delivery and implementation stages. Future efforts should prioritize not only technological innovation but also the development of integrated, patient-centered, and sustainable service models supported by robust implementation frameworks. These findings suggest that discussions of AI in mental health should extend beyond technological innovation to encompass structural transformation across the service system.

## Figures and Tables

**Table 1:** Search strategy overview for each database.

Database	Search Query	Filters/Limits
PubMed	((“mental health services” [Title/Abstract] OR “mental health care” [Title/Abstract] OR “psychiatric services” [Title/Abstract] OR “community mental health” [Title/Abstract]) AND (“artificial intelligence” [Title/Abstract] OR “machine learning” [Title/Abstract] OR “clinical decision support” [Title/Abstract] OR “generative AI” [Title/Abstract] OR “chatbot” [Title/Abstract]) AND (“service delivery” [Title/Abstract] OR implementation [Title/Abstract] OR practice [Title/Abstract] OR “case management” [Title/Abstract])) OR ((“Mental Health” [Mesh] OR “Mental Disorders” [Mesh] OR “mental health” [tiab] OR “behavioral health” [tiab]) AND (“Artificial Intelligence” [Mesh] OR “Machine Learning” [Mesh] OR “artificial intelligence” [tiab] OR “machine learning” [tiab] OR “deep learning” [tiab]) AND (“service*” [tiab] OR “implementation” [tiab] OR “practice*” [tiab] OR “workflow” [tiab]))	Language: English PUBYEAR >2015 AND PUBYEAR <2027
Scopus	(TITLE-ABS-KEY (“mental health service*” OR “mental health care” OR “psychiatric service*” OR “community mental health”)) AND (TITLE-ABS-KEY (“artificial intelligence” OR “machine learning” OR “generative AI” OR chatbot* OR “decision support system*”)) AND (TITLE-ABS-KEY (implementation OR “service delivery” OR workflow OR practice OR “case management”))	

**Table 2:** Extraction and Mapping Framework.

Dimension	Category (Label)	Definition and Examples
General Study Characteristics	Year	2015 < YEAR < 2027
	Study Design	Experimental, Pre-Post/Pilot, Observational, Qualitative/Mixed, Implementation/Usability
	Age Group	Child/Adolescent, Adult, Older Adult, Mixed/All
AI Technological Configuration	AI Type	Generative AI, ML Predictive, NLP, Decision Support, Multi-Modal
	Data Sensitivity	High (Clinical records, diagnosis, voice/image), Moderate (Self-report symptoms), Low (Aggregated)
Service Delivery Context	Service Function	Screening/Assessment, Treatment/Intervention, Case Management/Admin, Monitoring/Follow-up, Peer/Community Support
	Primary User	Clinician, Patient, Peer Worker, Manager/Admin
	Setting	Clinical, Community, Digital Only, Hybrid
	Implementation Stage	Pilot, Implemented (Real-world), Scale-up, Discontinued
	Study Focus	Effectiveness, Usability/Acceptance, Feasibility/Implementation
Diversity, Equity, and Bias Consideration *	Not reported (Level 0)	No mention of diversity or bias
	Mentioned (Level 1)	Conceptual discussion without analysis
	Evaluated (Level 2)	Empirical analysis of bias or subgroup differences

* Diversity, Equity, and Bias Consideration: Whether the study reported, discussed, or evaluated issues related to population diversity (e.g., race, gender, socioeconomic status) or potential bias in AI models.

**Table 3:** Summary of Characteristics of the Included Studies.

Author (Year)	AI Type	Setting	Primary User	Stage	Outcome
Stephenson et al. (2026) [7]	ML–predictive	Clinical	Clinician	Implemented	Reduced wait times; improved clinical outcomes
Dayal et al. (2025) [12]	ML–predictive	Clinical	Clinician	Pilot	Improved suicide risk identification; ethical concerns
Santisteban et al. (2025) [13]	Decision support	Clinical	Clinician	Implemented	High data collection and monitoring efficiency
Stroud et al. (2025) [14]	Decision support	Clinical	Clinician	Pilot	Identified benefits and risks of AI integration
Natale et al. (2025) [15]	Decision support	Community	Clinician	Pilot	Improved teaching practices and High Acceptability
Yu Wu et al. (2025) [16]	Multimodal	Clinical	Clinician	Implemented	Increased efficiency; reduced workload
Brandl et al. (2024) [17]	ML–predictive	Digital	Clinician	Pilot	High predictive accuracy; personalized support
Chang et al. (2024) [18]	NLP-based	Community	Patient	Implemented	High engagement and repeated use
Chiauzzi et al. (2024) [19]	Generative	Digital	Patient	Pilot	Significant reduction in depression/anxiety
Farrand et al. (2024) [20]	Generative	Digital	Patient	Implemented	High engagement; symptom improvement
Rollwage et al. (2023) [8]	Generative	Clinical	Patient	Implemented	Reduced wait time; improved recovery rates
Sadeh-Sharvit et al. (2023) [21]	Decision support	Clinical	Clinician	Implemented	Improved retention and symptom reduction
Mukherjee et al. (2022) [22]	Decision support	Community	Clinician	Pilot	Identified implementation barriers and facilitators
O’Callaghan et al. (2022) [23]	ML–predictive	Digital	Clinician	Implemented	High remission rates and satisfaction
Roebroek et al. (2022) [24]	Decision support	Clinical	Clinician	Implemented	Improved evidence-based decision-making
Tewari et al. (2021) [25]	Decision support; ML–predictive	Community	Clinician	Implemented	High screening and follow-up rate

**Table 4:** Distribution of AI service functions and primary user.

Service Function	Clinician	Patient	Peer Worker	Manager/Admin
Case management	9 (34.6%)	0 (0.0%)	3 (11.5%)	2 (7.7%)
Screening/Assessment	9 (34.6%)	0 (0.0%)	1 (3.8%)	1 (3.8%)
Treatment/Intervention	8 (30.8%)	5 (19.2%)	2 (7.7%)	0 (0.0%)
Monitoring/Follow-up	6 (23.1%)	2 (7.7%)	1 (3.8%)	1 (3.8%)

**Table 5:** Levels of Diversity, Equity, and Bias Consideration Across Included Studies.

Level	Definition	Description	Studies (*n* = 26)
Level 0	Not reported	No mention of diversity, equity, or bias; only basic demographic information reported without further discussion	Stephenson et al. (2026) [7]; Brandl et al. (2024) [17]; Chang et al. (2024) [18]; Farrand et al. (2024) [20]; Sibley et al. (2024) [26]; Sadeh-Sharvit et al. (2023) [21]; Almeda et al. (2022) [27]; Danieli et al. (2021) [28]; Sels et al. (2021) [5]; Tasma et al. (2018) [29]; Ally et al. (2016) [30]
Level 1	Mentioned	Conceptual discussion of bias, equity, or generalizability without empirical subgroup analysis	Dayal et al. (2025) [12]; Santisteban et al. (2025) [13]; Stroud et al. (2025) [14]; Wu et al. (2025) [16]; Patrickson et al. (2024) [31]; Creed et al. (2022) [32]; Mukherjee et al. (2022) [22]; O’Callaghan et al. (2022) [23]; Roebroek et al. (2022) [24]; Tewari et al. (2021) [25]; Oakey-Neate et al. (2020) [6]
Level 2	Evaluated	Empirical evaluation of bias or subgroup differences (e.g., gender, age, ethnicity)	Natale et al. (2025) [15]; Chiauzzi et al. (2024) [19]; Rollwage et al. (2022, 2023) [8,33]

## Data Availability

No new data were created or analyzed in this study. Data sharing is not applicable to this article.

## Abbreviations

The following abbreviations are used in this manuscript:

AI	Artificial Intelligence
DSS	Decision Support System
LLM	Large Language Model
ML	Machine Learning
NLP	Natural Language Processing

## Appendix A

**Table A1:** Summary of Characteristics of the Included Studies.

**Author (Year)**		**Age Group**	**Study Design**	**Setting**	**AI Type**	**Implementation Stage**
Stephenson et al. (2026)	[7]	Mixed	Observational	Clinical	ML–predictive AI	Implemented
Dayal et al. (2025)	[12]	Adult; Child/Adolescent	Qualitative/Mixed	Clinical	ML–predictive AI	Pilot
Santisteban et al. (2025)	[13]	Mixed	Implementation/Usability	Clinical	Decision support AI	Implemented
Stroud et al. (2025)	[14]	Mixed	Qualitative/Mixed	Clinical	Decision support AI	Pilot
Natale et al. (2025)	[15]	Child/Adolescent	Pre–post	Community	Decision support AI	Pilot
Wu et al. (2025)	[16]	Adult	Pre–post	Clinical	Multimodal AI	Implemented
Brandl et al. (2024)	[17]	Older adult	Qualitative/Mixed	Digital only	ML–predictive AI	Pilot
Chang et al. (2024)	[18]	Adult	Pre–post	Community	NLP-based AI	Implemented
Chiauzzi et al. (2024)	[19]	Adult	Pre–post	Digital only	Generative AI	Pilot
Farrand et al. (2024)	[20]	Adult	Observational	Digital only	Generative AI	Implemented
Patrickson et al. (2024)	[31]	Adult	Qualitative/Mixed	Hybrid	Decision support AI	Pilot
Sibley et al. (2024)	[26]	Child/Adolescent	Qualitative/Mixed	Community	Decision support AI	Pilot
Rollwage et al. (2023)	[8]	Mixed	Observational	Clinical	Generative AI	Implemented
Sadeh-Sharvit et al. (2023)	[21]	Adult	Experimental	Clinical	Decision support AI	Implemented
Almeda et al. (2022)	[27]	Adult	Observational	Community	Decision support AI	Implemented
Creed et al. (2022)	[32]	Adult	Qualitative/Mixed	Community	Decision support AI	Pilot
Mukherjee et al. (2022)	[22]	Mixed	Implementation/Usability	Community	Decision support AI	Pilot
O’Callaghan et al. (2022)	[23]	Mixed	Observational	Digital only	ML–predictive AI	Implemented
Roebroek et al. (2022)	[24]	Adult	Experimental	Clinical	Decision support AI	Implemented
Rollwage et al. (2022)	[33]	Mixed	Pre–post	Clinical	Generative AI	Implemented
Danieli et al. (2021)	[28]	Mixed	Experimental	Hybrid	All AI	Pilot
Sels et al. (2021)	[5]	Adult	Pre–post	Hybrid	Multimodal AI	Pilot
Tewari et al. (2021)	[25]	Adult	Qualitative/Mixed	Community	Decision support;	Implemented
					ML–predictive AI	
Oakey-Neate et al. (2020)	[6]	Mixed	Implementation/Usability	Community	ML–predictive AI	Pilot
Tasma et al. (2018)	[29]	Adult	Pre–post	Clinical	Decision support;	Pilot
					ML–predictive AI	
Ally et al. (2016)	[30]	Mixed	Pre–post	Clinical	Decision support AI	Implemented
**Author (Year)**		**Data Type**	**Service Function**	**Primary User**	**Study Focus**	
Stephenson et al. (2026)	[7]	Predictive analytics	Screening/Assessment	Clinician	Effectiveness	
Dayal et al. (2025)	[12]	Predictive analytics	Screening/Assessment	Clinician	Feasibility/Implementation	
Santisteban, J.A. et al. (2025)	[13]	Structured clinical data	Treatment/Intervention	Clinician	Feasibility/Implementation	
Stroud, A.M. et al. (2025)	[14]	Structured clinical data	Monitoring/Follow-up	Clinician; Manager	Feasibility/Implementation	
Natale, R. et al. (2025)	[15]	Structured clinical data	Screening/Assessment	Clinician	Usability/Acceptance	
Wu et al. (2025)	[16]	Multimodal	Case management;	Clinician	Feasibility/Implementation	
			Screening; Treatment			
Brandl, L. et al. (2024)	[17]	Predictive analytics	Monitoring/Follow-up	Clinician	Feasibility/Implementation	
Chang et al. (2024)	[18]	NLP-based	Peer support; Treatment	Patient	Feasibility/Implementation	
Chiauzzi, E. et al. (2024)	[19]	Generative	Treatment/Intervention	Patient	Effectiveness	
Farrand, P. et al. (2024)	[20]	Generative	Treatment/Intervention	Patient	Effectiveness; Usability	
Patrickson, B. et al. (2024)	[31]	Structured clinical data	Monitoring/Follow-up	Clinician	Feasibility/Implementation	
Sibley et al. (2024)	[26]	Structured clinical data	Case management	Clinician	Feasibility/Implementation	
Rollwage et al. (2023)	[8]	Generative	Screening/Assessment	Patient	Effectiveness	
Sadeh-Sharvit et al. (2023)	[21]	Structured clinical data	Case management	Clinician	Effectiveness; Usability	
Almeda, N. et al. (2022)	[27]	Structured clinical data	Case management	Manager	Feasibility/Implementation	
Creed, T.A. et al. (2022)	[32]	Structured clinical data	Case management	Clinician	Feasibility/Implementation	
Mukherjee, A. et al. (2022)	[22]	Structured clinical data	Screening/Assessment	Clinician; Peer worker	Feasibility/Implementation	
O’Callaghan, E. et al. (2022)	[23]	Predictive analytics	Treatment/Intervention	Clinician	Feasibility/Implementation	
Roebroek, L.O. et al. (2022)	[24]	Structured clinical data	Treatment/Intervention	Clinician	Feasibility/Implementation	
Rollwage, M. et al. (2022)	[33]	Generative	Screening/Assessment	Clinician	Effectiveness	
Danieli et al. (2021)	[28]	NLP-based	Treatment/Intervention	Patient	Effectiveness	
Sels, L. et al. (2021)	[5]	Multimodal	Monitoring/Follow-up	Patient	Feasibility/Implementation	
Tewari, A. et al. (2021)	[25]	Predictive analytics	Case management;	Clinician; Peer worker	Feasibility/Implementation	
			Screening; Treatment			
Oakey-Neate et al. (2020)	[6]	Predictive analytics	Monitoring/Follow-up	Clinician; Patient	Feasibility/Implementation	
Tasma, M. et al. (2018)	[29]	Predictive analytics	Treatment/Intervention	Clinician	Feasibility; Usability	
Ally, B.A et al. (2016)	[30]	Structured clinical data	Screening/Assessment	Clinician	Effectiveness	

**Table A2:** Summary of Service Interventions, Measurement Instruments, and Outcomes of Included Studies.

Author (Year)	AI Tool	Intervention	Service Users	Service Context	Measures	Outcomes
Stephenson et al. (2026) [7]	AI-assisted psychiatric triage system and therapist-assisted Online CBT	AI system to facilitate rapid psychiatric triage followed by therapist-guided online cognitive behavioral therapy	Outpatients and psychiatric providers	Clinical Setting Ambulatory Psychiatric	Wait times (referral to intake) Clinical outcomes (PHQ-9, GAD-7) Qualitative perceptions	Reduced Wait Times: 74.8% Clinical Improvement: Significant reductions in both depression and anxiety scores Provider high satisfaction
Dayal et al. (2025) [12]	ML suicide risk classification models applied to Electronic Health Records (EHR)	ML algorithms to identify at-risk youth and alerting providers via risk flags in the EHR	providers (*n* = 45), patients (*n* = 9), caregivers (*n* = 1)	Clinical Setting Pediatric ambulatory care	Risk model clinical preferences Usability perspectives Implementation barriers Implementation facilitators	Improve early identification of suicide risk Providers preferred imminent risk alerts and integrated “next-step” protocols Ethical issues included data privacy, patient autonomy, potential bias
Santisteban, J.A. et al. (2025) [13]	Brain Health Databank Electronic health records (EHR), and clinical decision support tools	Measurement-based care workflow using digital tools to collect patient-reported outcomes and clinical data for real-time clinician feedback	Patients (*n* > 3500 enrolled), Clinicians, and researchers	Clinical Setting Centre for Addiction and Mental Health (CAMH)	Patient enrollment rates Digital assessment completion rates Data integration efficiency Clinical dashboard usage	Enrollment: Over 3500 patients enrolled Data Collection: High completion rates for digital questionnaires (85%) Successfully integrated multi-modal data (clinical, digital, and potentially biological) into a unified databank for real-time clinical monitoring
Stroud, A.M. et al. (2025) [14]	Comprehensive Psychiatric AI Tools (prescribing aids, chatbot CBT, diagnostic assistants, and suicide risk predictors)	Physician Evaluation of AI Integration Exploration of physician perspectives on the potential impact of AI tools	Clinician 42 physicians	Clinical Setting Academic medical center	Interview (benefits, risks, and long-term impacts)	Benefits: drug efficacy, reduced administrative burnout, empowerment in decision-making Risks: Over unreliable recommendations, clinician dependence leading to skill erosion, increased data entry burden
Natale, R. et al. (2025) [15]	Jump Start on the Go (JS Go) A bilingual, AI-enabled Mental Health Consultation	A 14-week mobile adaptation JS consultation model delivering resources and strategies across four pillars: safety, behavior support, self-care, and communication	28 Early Care and Education teachers and 114 children	Community Setting	Teacher classroom practices Child psychosocial outcomes App usability and acceptability Semi-structured interviews	Teaching Practices: improving behavior support and resiliency practices. Child Outcomes: improvements in protective factors and a reduction in internalizing behaviors. High Acceptability: app as easy to use and helpful for reinforcing therapeutic strategies
Wu et al. (2025) [16]	Features facial emotion recognition, speech-to-text transcription, and automated summary generation	AI-supported remote intervention Provides real-time emotional analysis, automated session summaries	24 practitioners and 8 clients	Clinical Setting Real-world clinical trials and sandbox testing environments	Reduction in report writing time System usability ratings Task completion rates Emotion recognition accuracy Qualitative interviews	Efficiency: Reduced writing time over 50%. High Usability: Ease-of-use rated 4.83 by therapists and 4.33 by clients Technical Accuracy: 78.3% emotion recognition accuracy and 100% eye-tracking accuracy
Brandl, L. et al. (2024) [17]	AI Monitoring Tool Utilizes a fuzzy cognitive map decision algorithm and self-report monitoring questionnaires	A 10-week web-based intervention for older mourners, featuring biweekly mental health self-checks and AI-driven risk detection to guide users toward offline support	Older mourners (*n* = 44 for classification; *n* = 21 for regression) and 4 e-coaches	Community Setting Web-based e-mental health service	Classification performance Predictive value for clinical change (Grief, Depression, Loneliness scores) Qualitative e-coach experiences	High Accuracy: F1-score of 0.91 Predictive Value: significantly predicted changes in grief symptoms (*p* = 0.002) Perception: valued the tool for personalizing email guidance and detecting deterioration
Chang et al. (2024) [18]	Wysa (AI chatbot)	Self-guided AI support Rule-based chatbot conversations + Gratitude challenge	Health care workers	Community Setting workplace-based, within healthcare organization	Engagement rates Session frequency GAD-7, PHQ-2 User feedback	Engagement rate (80.1%) Avg. 10.9 sessions Strong repeat-use for sleep/anxiety tools
Chiauzzi, E. et al. (2024) [19]	Woebot for Mood and Anxiety (W-MA-02) A relational AI agent utilizing NLP	Text-based interface on a smartphone app providing goal-oriented conversations based on CBT, IPT, and DBT elements, including mood tracking	256 adults with depressive or anxiety symptoms	Community Setting A naturalistic sample recruited via social media (Facebook/Instagram)	PHQ-8 (depression) GAD-7 (anxiety) scores	Significant Symptom Reduction: Mean PHQ-8 score dropped by 7.28 and GAD-7 by 7.45
Farrand, P. et al. (2024) [20]	Iona Mind Well-being app An AI-led mental health app using NLP and a conversational agent	App-based Low-Intensity CBT (LICBT) Automated worry management modules including Cognitive Restructuring, Problem Solving, and Worry Time	1180 real-world users	Community Setting	Technique completion rates Session engagement PHQ-8 (depression) and GAD-7 (anxiety) scores	Engagement: Cognitive Restructuring was used most frequently (54.7%), followed by Problem Solving (26.6%) Completion: 76.5% of worry management sessions were completed to the end Clinical Efficacy: Significant reduction in depression (PHQ-8 mean diff = −2.5, *p* < 0.001) and anxiety (GAD-7 mean diff = −2.7, *p* < 0.001)
Patrickson B. et al. (2024) [31]	Includes a mobile self-monitoring app using Ecological Momentary Assessments	A consumer-centered model providing self-monitored assessments, real-time treatment recommendations, and psychoeducation tools to empower self-management	Adult ADHD (*n* = 9) healthcare practitioners (*n* = 6)	Community Setting Specialized adult ADHD care	Thematic barriers and enablers Acceptability of app features Usability of app features	Enablers: Empowerment through data insights, and customized mobile support, streamlined information exchange, transparency Barriers: Concerns regarding data privacy, technical overwhelm, administrative complexity
Sibley et al. (2024) [26]	Lyssn AI-based Motivational Interviewing integrity monitoring	STAND (Supporting Teens’ Autonomy Daily) Behavioral therapy blended with Motivational Interviewing (MI), digital clinician dashboard	ADHD and parents, community therapists (*n* = 6) agency supervisors (*n* = 3)	Community Setting Community mental health clinics	MI and content fidelity (via AI-generated metrics), Stakeholder feasibility, acceptability	Demonstrated initial feasibility and acceptability Promise in monitoring treatment quality without the need for constant expert oversight
Rollwage et al. (2023) [8]	Limbic Access A conversational AI chatbot AI-enabled self-referral and assessment support	The chatbot collects intake information (eligibility, demographics) and detailed clinical data (PHQ-9, GAD-7, etc.)	64,862 adult patients referred for psychological therapies	Clinical Setting England’s National Health Service (NHS)	Assessment duration (min) Wait times for assessment and treatment (days) Dropout rates (%) Treatment allocation accuracy (%) Reliable recovery rates (%)	Assessment Time: reduced by 12.7 min Wait Times: Reduced by 2.2 days for assessment and 5 days for treatment Dropout rates decreased and treatment allocation changes (step-ups/downs) were nearly halved Recovery Rate: Significantly higher (58%)
Sadeh-Sharvit et al. (2023) [21]	Eleos Health AI platform for behavioral health	The AI platform summarizes and transcribes sessions, provides feedback on the use of Evidence-Based Practices, and drafts progress notes	47 adults with depressive or anxiety disorders and mental health practitioners	Clinical Setting Community-based outpatient clinic	PHQ-9 (depression) GAD-7 (anxiety) Treatment attendance (retention) Progress note submission time	Symptom: 34% reduction in depression and 29% reduction in anxiety Retention: AI group attended 67% more sessions Efficiency: submitted notes 55 h earlier
Almeda, N. et al. (2022) [27]	Efficient Decision Support-Mental Health A hybrid tool combining Monte Carlo simulation, Data Envelopment Analysis, and a fuzzy inference engine	Performance Evaluation of Supported Accommodation Assessing the influence of seven care quality domains on the relative technical efficiency of three main types of supported accommodation services	Service managers and mental health accommodation services	Community Setting MH-supported accommodation services	Relative Technical Efficiency scores QuIRC-SA domains - living environment - therapeutic environment - treatments, Self-management - social interface - human rights - recovery-based practice	Heterogeneity: Technical efficiency for “move-on” residential services varied from 0.7 (baseline) to 0.86 Efficiency Gap: Supported housing services showed an average RTE baseline of 0.54, which rose to 0.64 with quality aggregation Negative Factor: Staying in services longer than the expected 2 years had a significant negative impact on performance
Creed, T.A. et al. (2022) [32]	AI-based CBT fidelity measurement tool An automated fidelity scoring supervision tool utilizing machine learning	Evaluation of the feasibility and acceptability of integrating automated fidelity scoring into community CBT supervision	18 community mental health therapists 12 agency leaders (supervisors/directors)	Community Setting Community mental health services	Self-reports of knowledge Acceptability, appropriateness, Feasibility of AI tools Qualitative feedback from discussions	High Acceptability: high scores for the tool’s acceptability and appropriateness Optimism gap: Agency leaders were significantly more optimistic about the implementation Role of AI: Participants emphasized that AI should function as a “support tool”
Mukherjee, A. et al. (2022) [22]	Mobile Decision Support System (mDSS) An electronic clinical decision support tool for healthcare workers	SMART Mental Health A complex intervention combining a community-based anti-stigma campaign and a mDSS-led screening and management program by primary care workers	Adult population (aged > 18) primary care doctors	Community Setting Rural primary health centers and villages	RE-AIM framework Fidelity Qualitative interviews Focus group discussions App usage logs	Identification of barriers and facilitators to implementation, process metrics (fidelity, reach), and mechanisms of intervention impact in LMIC settings
O’Callaghan, E. et al. (2022) [23]	Precision prescribing algorithm Clinical decision support tool for psychiatric medication	Using a data-driven algorithm to recommend initial psychiatric medications for depression and anxiety, followed by longitudinal outcome monitoring	3889 adult patients	Clinical Setting Telepsychiatry service (Brightside Health)	Feasibility (completion rates) Acceptability (patient satisfaction, alliance, and helpfulness scores) Clinical outcomes (PHQ-9 and GAD-7 scores) Remission rates at 12 weeks	High Feasibility: 92.4% of eligible participants received an algorithm-recommended prescription Positive Acceptability: Patients rated service satisfaction at 4.71/5 and algorithm helpfulness at 4.48/5 Clinical Efficacy: 54.3% depression remission and 55.4% anxiety remission rates by week 12
Roebroek, L.O. et al. (2022) [24]	TREAT (Treatment-E-Assist) A computerized clinical decision aid linking Routine Outcome Monitoring (ROM) data to evidence-based guidelines	TREAT-supported consultation vs. Treatment as Usual (TAU) Comparing clinical decision-making processes when clinicians use TREAT versus standard practice during consultations	Clinicians 166 patients	Clinical Setting Outpatient psychiatric care	Number of discussed care needs (psychiatric, physical, and social) The number of evidence-based treatment decisions made (via modified CDRC questionnaire)	Discussed Care Needs: Significant increase in TREAT group compared to TAU Evidence-based Decisions: Significant increase in decisions aligned with guidelines for the TREAT group compared to TAU Focus areas: Improved discussion specifically regarding physical health and social well-being
Rollwage, M. et al. (2022) [33]	Limbic Access Conversational AI chatbot	AI-enabled self-referral and triage The chatbot automates the collection of patient demographics and clinical data (PHQ-9, GAD-7)	58,475 patients mental health practitioners	Community Setting Psychological Therapies (IAPT) services	Reliable recovery rates (symptoms + below clinical cut-off) Economic cost-effectiveness (cost per additional recovery)	Improved Recovery: rose from 47.1% to 48.9% Cost-Effectiveness: Estimated cost of £103–£207 per additional recovery, which is significantly more efficient than hiring more staff (£1021).
Danieli et al. (2021) [28]	TEO (m-PHA: Mobile Personal Health Care Agent)	Blended Intervention 8 weekly CBT sessions via video + AI-led ABC note-taking support	Aging workers with stress, anxiety, or depression	Community (Remote) and Clinical Setting	SCL-90-R, PSS, OSI, MoCA	Significant trends in symptom reduction High therapist acceptance
Sels, L. et al. (2021) [5]	Mobile Coach platform An open-source system for Ecological Momentary Assessment and passive sensing, integrated with ML for risk prediction	SIMON Protocol (Suicide Monitoring and Prediction) 4 weeks of intensive monitoring (EMA 6 times/day + passive sensing via smartphone and wearable)	150 psychiatric patients	Clinical Setting Psychiatric hospitals and the subsequent transition to community care	Suicidal ideation (EMA) Physiological data (heart rate, activity, skin temperature) Smartphone logs (app use, communication) Clinical scales (BSS, PHQ-9, GAD-7)	Focused on establishing a personalized machine learning model for identifying real-time near-term predictors of suicidal ideation and facilitating early risk detection; currently, no empirical outcome measures are reported
Tewari, A. et al. (2021) [25]	Mobile Decision Support System (mDSS) An Android-based application for screening and clinical decision support	SMART Mental Health Project A multifaceted intervention combining a community-based anti-stigma campaign and a primary care-led screening and management program using mDSS	Community members (>18 years) Primary Care Physicians (PCPs) Community Health Workers (ASHAs)	Community Setting Rural villages	Feasibility Acceptability Screening rates Referral completion Follow-up rates Qualitative barriers/facilitators	Screening: 23,839 (97.7%) out of 24,402 Detection: 1327 (5.6%) individuals screened positive for depression and/or anxiety Referral: 241 (18.2%) individuals visited a PCP following referral Follow-up: 149 (61.8%) of those who visited a PCP had >1 follow-up visit Qualitative: High acceptability; barriers included drug shortages and lack of specialists
Oakey-Neate et al. (2020) [6]	AI2 A machine learning-based personal nudging system and clinical decision support tool	Real-time monitoring and alerting Analyzing national Medicare data to detect medication non-adherence or missed health checks	Patients and community mental health professionals	Community Setting Community mental health clinics and primary care settings	Responsiveness to alerts Proportion of actioned alerts Usability and Acceptability	Focus on identifying the implementation barriers and enablers to validate predictive models and conduct robust, large-scale RCTs in clinical settings
Tasma, M. et al. (2018) [29]	Treatment-E-Assist (TREAT) A computerized clinical decision aid (CDA) linking diagnostic data to clinical guidelines	Implementation of TREAT system Automating the link between Routine Outcome Monitoring data and the Multidisciplinary Guideline for Schizophrenia to provide personalized treatment advice	6 clinicians and 16 patients with psychotic disorders	Clinical Setting Outpatient Functional Assertive Community Treatment (FACT) teams at a psychiatric institute	Feasibility, Acceptability Ease of use, Usefulness Clinicians’ experiences Qualitative interviews	General Satisfaction: an average of 7.5/10 and its integration with ROM at 7.3/10 Ease of Use: Mean score of 4.33/5 for ease of use and 4.33/5 for requiring little mental effort Future Intent: 4.83/5 on intention to actively use Time Efficiency: 5–15 min to > 30 min
Ally & Stallman (2016) [30]	Clozapine Decision Support Tool (CDST) A multidisciplinary, Brief guideline-based tool	m-Implementation of CDST Clozapine Initiation Pathway to guide prescribing, dispensing, and ongoing monitoring	Patients (*n* = 67) and clinicians (doctors, nurses)	Clinical Setting Mental health facility	Physical health, ECG, hematology Prescribing accuracy	Significant improvements in monthly physical health monitoring, 6-monthly ECG monitoring Monitoring of waist circumference and bowel movements increased to 100% Improved consistency in medication charts regarding route and duration
